# Effects of Propeller and Stirring‐Speed on the Rheological, Physicochemical, and Sensory Characteristics of Creamed Honeys

**DOI:** 10.1111/jtxs.70059

**Published:** 2026-01-16

**Authors:** Letícia Oliveira Almeida Silva, Júlia Flávia Souza Andrade, Yasmim Emanuele Oliveira Marques, Janaina Mota Fiuza da Silva, Naiara Barbosa Carvalho, Robledo de Almeida Torres Filho, Vanelle Maria da Silva

**Affiliations:** ^1^ Universidade Federal de Viçosa Campus Florestal Instituto de Ciências Exatas e Tecnológicas Florestal Brazil

**Keywords:** controlled crystallization, dyce method, elastic modulus, purchase intention, sensory acceptance, thixotropy, viscosity, viscous modulus

## Abstract

Creamed honey has high added value. During its processing, the mixing conditions of macerated crystal seeds with liquid honey can influence crystallization and product creaminess. Therefore, the effects of propeller type (Naval and ARA‐S) and stirring speed (180 and 360 rpm) in a mechanical stirrer on the rheological, physicochemical, and sensory properties of creamed honey were evaluated. The physicochemical characteristics of the products complied with the limits established by the Codex Alimentarius. The creamed honeys exhibited typical pseudoplastic flow curves (*R* ^2^ > 0.9997 and RMSE < 12.81), with higher apparent viscosity at 10 s^−1^ (*η*_10_) (*p* < 0.05) for samples stirred with the Naval propeller (42.54 ± 1.84 Pa s) compared with those stirred using ARA‐S (36.30 ± 1.94 Pa s). The temperature dependence of *η*_10_ was described by the Arrhenius model (*R* ^2^ > 0.9978 and RMSE < 1.35). All treatments presented thixotropic behavior, fitted to the Weltman model (*R* ^2^ > 0.9632 and RMSE < 60.11), with no significant differences (*p* > 0.05) among treatments in terms of hysteresis area. Oscillatory rheological data revealed a liquid‐like behavior (viscous modulus (*G*″) > elastic modulus (*G*′)) for the creamed honeys. Both *G*′ and *G*″ were significantly affected (*p* < 0.05) by the interaction between propeller type and stirring speed. Temperature sweep tests revealed the low thermal stability of the crystalline structure in all treatments. Creamed honey showed high sensory acceptance (≥ 7.32) and purchase intent (≥ 3.78), with no significant differences (*p* > 0.05) among treatments, and superior (*p* < 0.05) scores compared with naturally granulated honey. Overall, these findings offer honey producers practical guidance to optimize processing and enhance sector profitability by supporting the commercialization of a higher‐quality, value‐added product.

## Graphical abstract

Mixing conditions influenced the rheological behavior of creamed honey, particularly viscosity and viscoelastic moduli. Despite structural sensitivity to temperature, all samples showed high sensory acceptance and purchase intent, highlighting processing parameters as critical for optimizing industrial production of creamed honey.

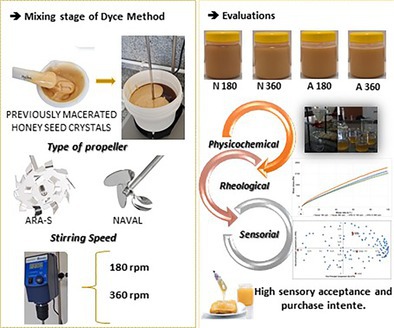

## 1 Introduction

Honey is a natural sweetener with high nutritional value, and its physicochemical, rheological, and sensory properties depend on various factors including harvesting methods, processing and storage conditions, floral source, and climate (Turhan et al. 2008; Venturini et al. 2007). It is produced from flower nectar and the secretions of certain plant parts, or plant‐sucking insect excretions (Venturini et al. 2007). Among the various uses of honey in food, it stands out as a phytotherapeutic agent and is used in traditional medicine (Mendes et al. 2009). Its nutritional and medicinal benefits are closely linked to its composition (Oliveira and Berto 2017), mainly comprising water, glucose, fructose, sucrose, and maltose, as well as mineral salts, vitamins, enzymes, proteins, and acids (Araújo et al. 2006).

Despite these characteristics, annual honey consumption in Brazil remains low at only 60 g per capita, compared to the global average of 240 g per capita (ABELHA, Associação Brasileira de Estudos das Abelhas 2025; Faeb 2022). Nevertheless, Brazil ranks eighth in global honey production (FAO 2023). In 2024, the national honey production value reached R$ 1,010,040,000.00 in 2024, with a total production of 67,313,986 kg (IBGE 2024), of which 37,890,000 kg were exported (ABEMEL 2024). Thus, there is a need in the country to develop and produce new honey‐derived products that combine healthiness, convenience, and sustainability—trends in the current food market—in order to increase per capita consumption and promote the growth of the beekeeping sector. These consumption and market gaps highlight the opportunity to add value through differentiated honey products such as creamed honey.

After honey is harvested, physicochemical and sensory changes continue to occur (Araújo et al. 2006). Natural crystallization may lead to the formation of few or numerous α‐d‐glucose monohydrate crystals in the product, negatively affecting consumer acceptance (Piana et al. 2023). Furthermore, consumers often associate this phenomenon with adulteration (Venturini et al. 2007). Water and sugar contents are the key factors influencing the rate of honey crystallization. The glucose‐to‐water ratio (G/W) is considered the best predictor of this crystallization tendency. Values below 1.7 indicate a low tendency to crystallize, whereas values above 2.0 suggest a rapid crystallization tendency (Lupano 1997; Sereti et al. 2021).

A controlled crystallization process was employed to produce creamed honey. This method has been proposed by Dyce (1975) and results in small, uniform crystals within the product, which confer good spreadability and a smooth, creamy texture without the perception of crystals in the mouth. Creamed honey stands out for its versatility and is commonly consumed with fruits and cheeses or applied to bread, pancakes, and toast (Piana et al. 2023; Suriwong et al. 2021), especially in European countries. In Brazil, this product remains relatively unknown but represents a potential alternative to boost the honey sector and add value to the product, particularly because it can be made from honey with a high tendency to crystallize, which is typically considered to have a lower market value.

Among the processing steps, crystallization temperature has a major influence on the final creaminess of the product. Studies indicate that 14°C is the optimal temperature for achieving the highest creaminess (Ozmen et al. 2025; Meixner et al. 2022; Costa et al. 2015). Another critical step involves the incorporation of previously prepared seed crystals into liquid honey. At this stage, the type of device used, stirring time, and mixing interval can affect the final product quality by influencing air incorporation and the number and size of crystals formed (Meixner et al. 2022). Several authors have described the use of different stirring devices during this stage, such as manual stirring with a spatula (Suriwong et al. 2020), mixers (Elhamid and Abou‐shaara 2016), helical propellers (Tappi et al. 2019, 2021), manual bucket stirrers, helical stirrers, and screw agitators (Meixner et al. 2022). Different stirring speeds have also been reported, such as 60 (Karahan et al. 2023), 0, 180, 360, and 540 rpm (Anina and Nirmal 2022; Costa et al. 2015), which influence the crystallization kinetics.

The development of the production process, considering factors such as honey composition, operational conditions, and storage parameters, is essential, as these aspects influence the size of the crystals formed in creamed honey and, consequently, its rheological and sensory characteristics (Ozmen et al. 2025; Meixner et al. 2022; Sereti et al. 2021; Tappi et al. 2021). Since honey is a food fluid, understanding its rheological behavior is of great interest to beekeepers and the honey industry, as it prevents unnecessary financial losses by allowing proper equipment sizing—such as mixers, pumps, and filling machines—and ensures adequate process and product quality control (Turhan et al. 2008). Creamed honey exhibits thixotropic non‐Newtonian behavior; its flow behavior depends on the duration of the applied shear rate, as described by the Weltman model (Karasu et al. 2015). Moreover, it may present a flow curve characteristic of the power‐law model due to its shear‐thinning (pseudoplastic) behavior (Karasu et al. 2015). In contrast, raw honey generally displays Newtonian behavior (Faustino and Pinheiro 2021).

To the best of our knowledge, no studies have reported the effects of homogenization conditions—specifically, the incorporation of previously prepared seed crystals into liquid honey—on the rheological properties of creamed honey, nor the relationship between rheological properties and sensory acceptance. Ozmen et al. (2025) highlighted the importance of exploring the effects of seed blends and processing variables to improve creamed honey quality.

Furthermore, no studies have investigated the processing of creamed Brazilian *Apis mellifera* honey. Therefore, evaluating operational parameters may provide valuable insights for beekeepers and the honey industry, encouraging the increase of per capita honey consumption in Brazil through the commercialization of a high‐quality, value‐added product. Hence, the objective of this study was to evaluate the effects of propeller type and stirring speed on the physicochemical, rheological, and sensory characteristics of creamed Brazilian *Apis mellifera* honey.

## 2 Materials and Methods

This study was approved by the Research Ethics Committee for Human Subjects under the registration number CAAE 60838322.2.0000.5153.

### 2.1 Experimental Design

The experiment used a completely randomized design (CRD) with a 2 × 2 factorial arrangement. The factors considered included agitation speed (180 and 360 rpm) and type of stirring propeller (Naval propeller and ARA‐S propeller), resulting in four treatments: N180, N360, A180, and A360, each with three replicates. The crystallized honey used as the raw material was also analyzed in addition to the other treatments.

### 2.2 Processing of Creamed Honey

Honey samples were collected in Espera Feliz (MG, Brazil). Creamed honey was processed as described by Dyce (1975), with a few modifications described by Krell (1996). The homogenization step was performed using a mechanical stirrer (model AE‐70, Gehaka) equipped with an ARA‐S or Naval propeller operating at 180 or 360 rpm to homogenize 4.0 kg of honey. Stirring was performed in four 20‐min sessions spaced 2 h apart. Approximately 7.5% of the formulation comprised previously macerated honey seed crystals. During homogenization, the temperature of the mixture was monitored using a thermocouple (model UT320, UniT) and maintained below 24°C. After homogenization, creamed honey was poured into 300 mL plastic jars and stored in a BOD incubator (LimaTec LT320T) at 14°C for 10 days (Krell 1996).

### 2.3 Physicochemical Analyses

Physicochemical analyses of honey were performed according to the standards established by Normative Instruction No. 11 (Brasil 2000). Analyses of diastatic activity, iodine reaction, acidity, insoluble solids, and hydroxymethylfurfural were performed following the methodologies described by the Adolfo Lutz Institute (2008). pH was measured according to Abu‐Jdayil et al. (2002) using a 10% (w/v) honey solution and a digital pH meter (HANNA HI 99163). Water activity was measured using a Testo 650 analyzer at 25°C. Color was evaluated using a colorimeter (Delta Vista 650G) employing the CIELAB system, illuminant D65, a 10° observer angle, and RSE. A bench digital refractometer (Ionlab RED‐D‐301) was used to determine the moisture content.

The contents of reducing sugars, total sugars, and apparent sucrose were determined using the Lane‐Eynon method (Carvalho et al. 2002; Adolfo Lutz Institute 2008).

Glucose and fructose contents were evaluated in raw honey using high‐performance liquid chromatography (HPLC) on a Shimadzu chromatograph equipped with a quaternary pump (LC‐20AT), diode array detector (SPDM‐20A), degasser (DGU‐20A5), interface module (CBM‐20A), and autosampler (SIL‐20A). The separations were performed using a Supelcogel 8H column (300 mm × 7.8 mm, cat. 59246‐U) and a pre‐column of Supelcogel 8H (10 mm × 7.8 mm). Elution was performed in an isocratic system, employing a mobile phase consisting of a phosphate buffer solution of KH_2_PO_4_ at 0.005 mol/L (pH = 2.7). The flow rate was set to 0.5 mL/min, with the column maintained at 30°C. The injection volume for both samples and standards was 20 μL, and detection was performed using a refractive index detector (Campos et al. 1999). Compounds were identified by comparing sample retention times with those of analytical standards (Sigma‐Aldrich, HPLC‐grade fructose and glucose) and quantified using external standard calibration.

### 2.4 Rheological Analysis

A Haake Mars IQ Air stress‐ and strain‐controlled oscillatory and rotational rheometer (Thermo Scientific Inc., Germany) equipped with a 35 mm diameter steel parallel plate and a 1 mm gap, as well as a Peltier temperature control system, was used to evaluate the steady and dynamic rheological properties of the creamed honey samples.

Steady shear rheology was investigated over a shear rate range of 0.1–100 s^−1^ through three consecutive cycles (upward, downward, and upward again) at 20°C to eliminate thixotropic effects and obtain a hysteresis loop. The relationship between shear rate and shear stress from the third cycle was evaluated using the Ostwald–de Waele and Herschel–Bulkley models. Temperature sweep tests of apparent viscosity (*η*_10_) were conducted at a constant shear rate of 10 s^−1^, within the temperature range of 14°C–30°C, using a heating rate of 1°C/min. The resulting *η*_10_ values were fitted to the Arrhenius equation to describe the temperature dependence of viscosity. The time‐dependent rheological behavior of creamed honey was also evaluated at a constant shear rate of 10 s^−1^ over a time range of 0–10 min, at 14°C and 20°C. The experimental data were then fitted to the Weltman model.

An amplitude sweep test was performed over a strain range of 0.01%–10% at 1 Hz to determine the linear viscoelastic region (LVR). Subsequently, a frequency sweep test was carried out within the LVR at 0.1% strain, over an angular frequency (*ω*) range of 0.1–10 Hz, at 20°C. A temperature sweep test was also conducted from 14°C to 30°C and back to 14°C, at a heating/cooling rate of 1 K/min and a frequency of 1 Hz, as described by Smanalieva and Senge (2009). During these tests, the storage modulus (*G*′), loss modulus (*G*″), loss tangent (tan *δ*), and complex viscosity (*η*)* were recorded. The dependence of *G*′, *G*″, and η* on angular frequency was modeled using a power–law relationship. All rheological properties were calculated using the Haake RheoWin Data Manager, version 4.95.0000 (Thermo Scientific, Germany).

### 2.5 Sensory Analysis

For sensory testing, 142 regular honey consumers aged over 18 years were recruited to evaluate the four treatments, and the raw material was naturally crystallized.

Sensory evaluations were conducted in individual booths under white‐light conditions. During the evaluations, the participants received a tray containing a coded sample in a plastic cup (50 mL) containing approximately 3 g of the sample in a balanced, random, and monadic order. Between different samples, the participants rinsed their mouths with water and a 1‐min rest interval was provided before presenting the next sample.

Consumers were provided evaluation sheets for each sample to record their acceptance of and purchase intentions for the product. Sensory acceptability in relation to appearance, flavor, texture, and overall impression was assessed using a nine‐point hedonic scale (1 = extremely dislike, 9 = extremely like). Subsequently, purchase intention was assessed using a five‐point purchase intention scale (1 = certainly would not buy; 5 = certainly would buy) (Minim 2018).

### 2.6 Statistical Analysis

Statistical analyses were performed using the statistical package for the social sciences (SPSS) software through analysis of variance (ANOVA) at a 5% significance level (*α* = 0.05) and descriptive statistics (mean, standard error of the mean, and coefficient of variation).

## 3 Results and Discussion

### 3.1 Physicochemical Characterization of Liquid Honey

All physicochemical parameters of the liquid honey complied with the regulatory limits (Brasil 2000; Codex 2001): diastase activity (29.24 ± 2.57 > 8°Goethe), free acidity (29.44 ± 0.78 < 50 meq/kg), pH (4.52 ± 0.09 < 5.50), water activity (aw 0.643 ± 0.004), hydroxymethylfurfural (HMF) content (3.44 ± 0.82 < 60 mg/kg), moisture content (18.57 ± 0.17 < 20.00%), insoluble solids (0.01 ± 0.01 < 1.00%), reducing sugars (69.24 ± 0.18 > 65.00%), total sugars (70.89 ± 0.28), apparent sucrose (1.56 ± 0.16 < 6.00%), and negative Lugol reaction, indicating the absence of adulteration as well as proper harvesting and processing.

Liquid honey exhibited a dark color (L* = 30.29), high chroma (C* = 24.38), a yellowish hue (b* = 23.10; *h* = 70.34), and a slightly reddish tone (a* = 7.82), aligned with the aforementioned regulations stating that honey color may range from colorless to dark brown.

The raw material exhibited an average glucose‐to‐fructose ratio of 1.25, corresponding to an intermediate crystallization tendency, that is between slow crystallization (> 1.33) and rapid crystallization (< 1.11) (Escuredo et al. 2014; Ma et al. 2016). The mean glucose (32.72%) and fructose (40.81%) levels were consistent with those reported previously. de Palomo‐León et al. (2023) reported glucose concentrations ranging from 25.6% to 37.3%, whereas Fadhil and Toamah (2023) reported fructose levels ranging from 36.23% to 40.18%.

### 3.2 Physicochemical Characterization of Creamed Honey

The physicochemical attributes of the creamed honey samples (Table 1) also met the requirements of Brazilian and international standards (Brasil 2000; Codex 2001). Overall, the creamed honey samples were light‐colored (L* 43.11), intense (C* 18.06), yellowish (b* 17.92; *h*° 82.92), and slightly reddish (a* 2.24). These values agree with both national and international legislation, which states that honey can range from colorless to dark brown (Brasil 2000; Codex 2001). Compared with liquid honey, creamed honey appeared lighter, less yellow, less red, and less intense. These findings corroborate those of Dettori et al. (2018), who reported increases in L* and decreases in h° during honey storage; these changes were attributed to the formation of glucose crystals that reflect light, enhancing brightness while reducing yellow components, evidencing the physical effects of crystallization on product appearance.

**TABLE 1:** Physicochemical properties of creamed honeys produced using a mechanical stirrer with different propeller types (Naval and ARA‐S propeller) and stirring speed (180 and 360 RPM) (*n* = 3).

Characteristics^1^	Shaker^2^		Speed (RPM)^2^		Overall average
	Naval propeller	ARA‐S propeller	180	360	
Hydroxymethylfurfural (mg/kg)	7.64 ± 2.76	8.14 ± 1.64	6.80 ± 2.01	8.98 ± 2.42	**7.89** ± **1.53**
Diastasis activity (*Gother*)	19.83 ± 2.24	14.77 ± 2.47	14.69 ± 1.71	19.90 ± 2.84	**17.30** ± **1.76**
Free acidity (meq/kg)	26.96 ± 1.15	28.74 ± 1.03	27.73 ± 1.25	27.97 ± 1.07	**27.85** ± **0.78**
pH	**4.55** ± **0.01** ^***a***^	**4.40** ± **0.02** ^***b***^	4.47 ± 0.03	4.48 ± 0.04	4.48 ± 0.02
*a*_*w*_	0.635 ± 0.002	0.628 ± 0.005	0.632 ± 0.003	0.632 ± 0.005	**0.632** ± **0.003**
Water (%)	**18.59** ± **0.10** ^***a***^	**18.37** ± **0.06** ^***b***^	18.58 ± 0.09	18.38 ± 0.09	18.48 ± 0.07
Minerals (%)	0.422 ± 0.019	0.412 ± 0.009	0.413 ± 0.010	0.420 ± 0.018	**0.417** ± **0.010**
Reducing sugars (%)	69.81 ± 0.31	71.51 ± 0.34	70.87 ± 0.65	70.46 ± 0.25	70.66 ± 0.34
Total sugars (%)	71.79 ± 0.37	72.74 ± 0.30	72.51 ± 0.37	72.02 ± 0.39	**72.26** ± **0.27**
Apparent sucrose (%)	1.89 ± 0.34	1.16 ± 0.29	1.54 ± 0.34	1.52 ± 0.37	**1.53** ± **0.24**
*Lugol* reaction	Negative	Negative	Negative	Negative	**Negative**
L*	**41.50** ± **1.26** ^***b***^	**44.73** ± **0.41** ^***a***^	42.96 ± 0.94	43.27 ± 1.38	43.11 ± 0.80
a*	2.16 ± 0.25	2.31 ± 0.17	2.27 ± 0.28	2.21 ± 0.12	**2.24** ± **0.15**
b*	17.69 ± 0.86	18.15 ± 0.51	17.94 ± 0.76	17.91 ± 0.67	**17.92** ± **0.48**
C*	17.83 ± 0.89	18.30 ± 0.52	18.09 ± 0.79	18.04 ± 0.68	**18.06** ± **0.50**
*H*	83.12 ± 0.45	82.73 ± 0.50	82.88 ± 0.64	82.97 ± 0.23	**82.92** ± **0.33**

*Note: p*(*F*): probability of the *F*‐test from the Analysis of Variance (ANOVA). Significance level (*p < 0.05)* in bold.

^1^ Means ± Standard Error.

^2^ Means followed by different letters (a‐b) differ from each other, within each factor (propeller type and stirring speed) according to the *F*‐test at the 5% significance level.

Only the L* values were significantly affected (*p* < 0.05) by the propeller type, with the ARA‐S propeller producing lighter creamed honey (*p* < 0.05); however, the other color indices were not influenced (*p* > 0.05) by the propeller design or stirring speed. Agitation using the ARA‐S propeller also yielded creamed honey with lower pH and moisture content (*p* < 0.05). The interaction between the propeller and stirring speed influenced the reducing sugars, with the lowest values (*p* < 0.05) obtained with the N180 treatment.

The HMF content, diastase activity, free acidity, water activity, mineral content, total sugars, and apparent sucrose in the creamed honeys were not significantly affected (*p* > 0.05) by the propeller type or stirring speed.

The low HMF content observed in creamed honey indicates the preservation of quality during processing. Furthermore, when comparing diastase activity, aw, and free acidity of the raw material (17.30°G, 0.632, and 27.85 meq/kg, respectively) with those of the processed product, no loss of enzymatic activity, stability, or evidence of fermentation was found. These results agree with those of previous studies demonstrating that, when creamed honey is produced from high‐quality honey under controlled conditions, the physicochemical quality indicators of the final product remain unaltered (Shlyamina et al. 2024).

### 3.3 Rheological Analysis of Creamed Honey

#### 3.3.1 Steady Shear Properties

**FIGURE 1:**
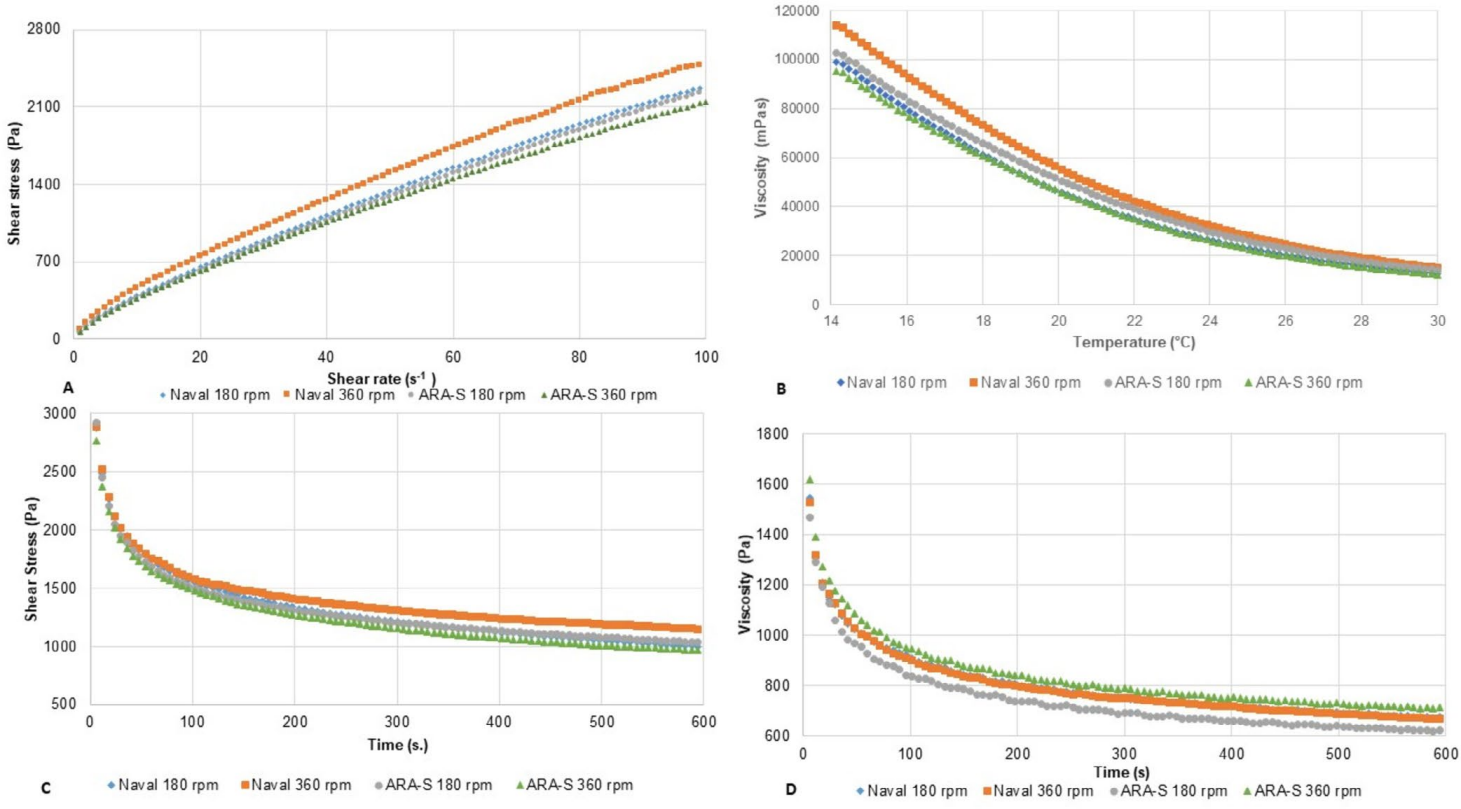
Relationship between shear stress and shear rate at 20°C (A); apparent viscosity as a function of temperature at a constant shear rate of 10 s^−1^ (B); and shear stress as a function of time at a constant shear rate of 10 s^−1^ at 14°C (C) and 20°C (D) (*n* = 3).

The rheological model that best represented the flow behavior of the samples (Figure 1) was the Power Law model (*R* ^2^ > 0.9997 and RMSE < 12.81) because of its lower complexity and because the statistical parameters *R* ^2^ and RMSE did not vary significantly among the evaluated models. The consistency index and flow behavior index varied, respectively, from 78.4 ± 0.66 Pa s^*n*^ and 0.75 for N360 to 55 ± 0.25 Pa s^*n*^ and 0.81 for A180 (Table 2). In a study conducted by Karasu et al. (2015), creamed honey exhibited pseudoplastic fluid characteristics, with *n* values ranging from 0.76 to 0.81. The shear‐thinning (pseudoplastic) flow behavior, also reported by Ozmen et al. (2025), is attributed to the controlled crystallization process of honey, which makes it more solid and consequently increases its resistance to flow (Schiassi et al. 2021).

**TABLE 2:** Rheological parameters of creamed honeys produced using a mechanical stirrer with different propellers (Naval and ARA‐S) and stirring speed (180 and 360 rpm), according to the Ostwald–de Waele model (*n* = 3).

Treatment^a^	*K*	*n*	*R* ^2^	RMSE	*η* (10 s^−1^) (Pa s)^b^
N180	63.0 ± 0.40	0.78 ± 0.001	0.9998	8.4	39.30 ± 2.01
N360	78.4 ± 0.66	0.75 ± 0.002	0.9997	12.8	45.77 ± 1.56
A180	55.0 ± 0.25	0.81 ± 0.001	0.9999	5.7	36.14 ± 1.50
A360	60.1 ± 0.25	0.78 ± 0.001	0.9999	5.3	36.47 ± 4.08

*Note: K*: consistency index (Pa s^*n*^); *n*: flow behavior index (dimensionless); *σ*_0_: yield stress; *η*: apparent viscosity (Pa s) rpm; *R* ^2^ : coefficient of determination; RMSE: root mean square error; N180: Naval propeller at 180 rpm; N360 = Naval propeller at 360 rpm; A180: ARA‐S propeller at 180 rpm; A360: ARA‐S propeller at 360 rpm.

^a^ Means ± Standard Error.

^b^ Speed: *p* > 0.05, propeller: *p* < 0.05, and interaction: *p* > 0.05.

The apparent viscosity (10 s^−1^) (*η*_10_) was affected (*p* < 0.05) (Table 2) only by the propeller type, with the Naval (42.54 ± 1.84 Pa s) showing higher apparent viscosity than that obtained with ARA‐S (36.30 ± 1.94 Pa s). The *η*_10_ of the samples decreased with increasing temperature (Figure 1). This effect is attributed to the higher molecular motion at elevated temperatures, which reduces intermolecular forces, thereby decreasing the *η*₁₀ (Jiang et al. 2021). The activation energy (Ea, kJ mol^−1^) of the creamed honeys, determined using the Arrhenius model (*R* ^2^ > 0.9978 and RMSE < 1.35), ranged from 90.43 (A180) to 96.03 kJ mol^−1^ (N180). Higher Ea values indicate a more organized molecular structure with stronger intermolecular bonds, which hinder molecular movement and require more energy to initiate flow. Such structures are also more stable and less prone to changes like phase separation (Rao et al. 2007). Karasu et al. (2015) reported an Ea of 36.62 kJ·mol^−1^ for the apparent viscosity of creamed honey at 50 s^−1^. The differences observed in the present study may thus be related to the honey composition and processing methods.

The decrease in *η*₁₀ under constant shear confirmed the thixotropic behavior of all treatments at 14°C and 20°C (Figure 1), owing to structural breakdown. At 20°C, a faster reduction in *η*_10_ was observed (Figure 1), as intermolecular forces tend to weaken at higher temperatures, thus facilitating disruption (Faustino and Pinheiro 2021). Therefore, *η*₁₀ stability during storage would be higher at 14°C than at 20°C.

The Weltman model was suitable for describing the shear stress versus time variation (*R* ^2^ > 0.9632 and RMSE < 60.11) of the treatments. The model constants (Table 3)—yield stress (A) and structure breakdown rate (B)—showed little variation among the treatments. The thixotropic hysteresis area was not affected (*p* > 0.05) by the propeller type or agitation speed (Table 3), indicating no significant differences among the treatments regarding structural breakdown and energy dissipation when subjected to stress (Rao et al. 2007; Karasu et al. 2015).

**TABLE 3:** Mean values of parameters A and B according to the Weltman model for creamed honeys produced using a mechanical stirrer with different propellers (Naval and ARA‐S) and stirring speed (180 and 360 rpm) (*n* = 3).

Treatment	Hysteresis area (Pa s)^b^	Time‐dependent behavior			
		14°C		20°C	
		A^a^	B^a^	A^a^	B^a^
N180	46796.7 ± 3986.32	3241.2 ± 23.5	−355.3 ± 4.3	1651.9 ± 14.0	−156.5 ± 2.5
N360	46200.0 ± 3426.58	3091.5 ± 33.7	−311.2 ± 6.1	1653.5 ± 14.1	−157.5 ± 2.6
A180	51370.0 ± 5153.76	3094.1 ± 35.9	−329.9 ± 6.5	1594.3 ± 16.0	−156.8 ± 2.9
A360	56530.0 ± 670.89	3073.7 ± 25.1	−335.9 ± 4.6	1735.9 ± 16.2	−165.0 ± 2.9

*Note:* A: yield stress limit (dimensionless); B: structural breakdown rate (dimensionless); *R* ^2^: coefficient of determination; RMSE: root mean square error. N180: Naval propeller at 180 rpm; N360: Naval propeller at 360 rpm; A180: ARA‐S propeller at 180 rpm; A360: ARA‐S propeller at 360 rpm.

^a^ Means ± Standard Error.

^b^ Speed: *p* < 0.01, propeller: *p* < 0.01, and interaction: *p* = 0.053.

#### 3.3.2 Dynamic Shear Properties

**FIGURE 2:**
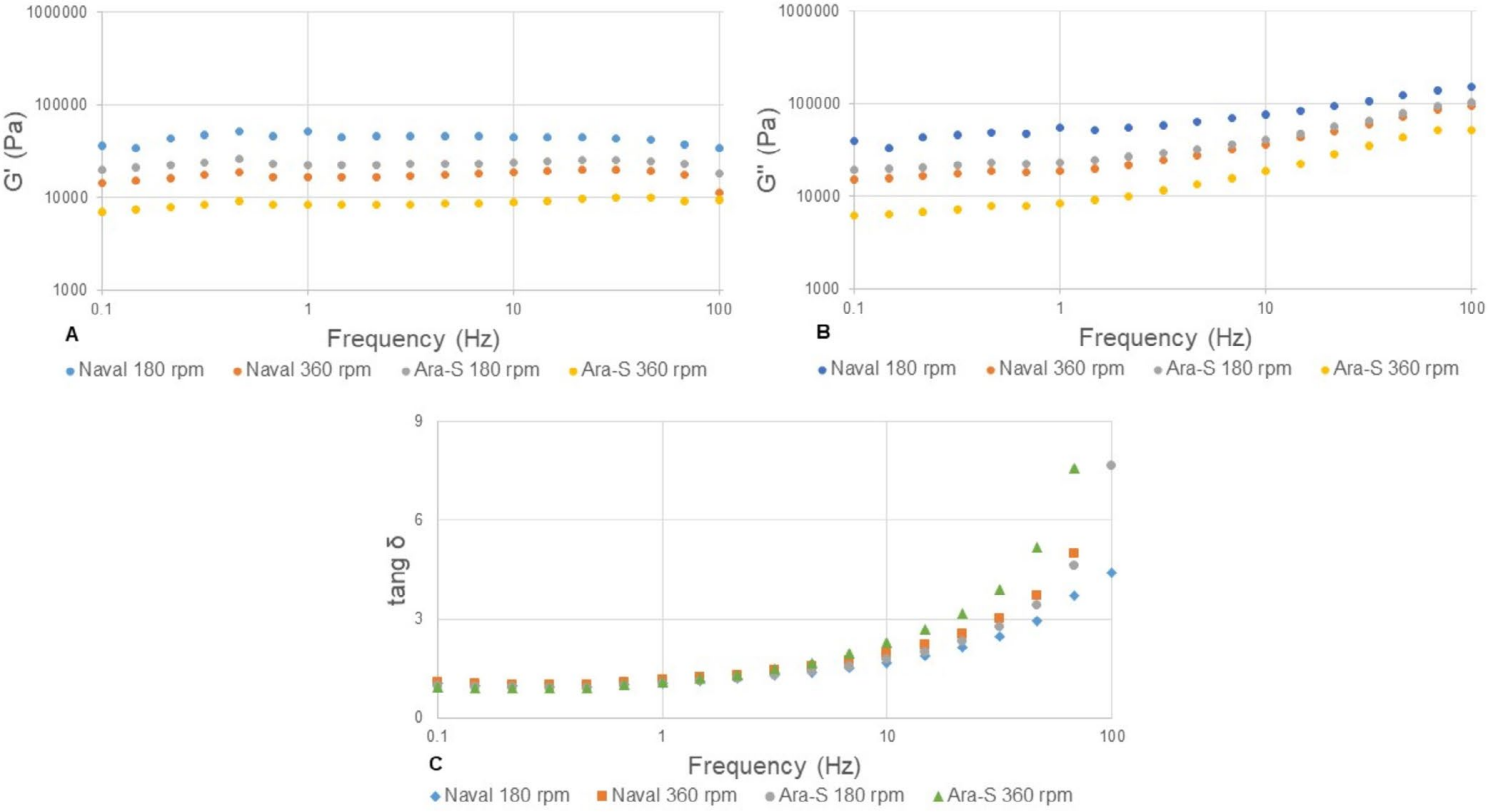
Frequency sweep behavior of the storage modulus (*G*′) (a), loss modulus (*G*″) (b), and phase tangent (tan *δ* = *G*″/*G*′) (c) of creamed honeys produced using different propeller types and stirring speeds at 20°C (*n* = 3).

The linear viscoelastic region of the samples was obtained at 0.1% strain. The behavior of the storage modulus (*G*′), loss modulus (*G*″), and loss tangent (tan *δ*) as a function of frequency (Figure 2a–c) showed that the creamed honeys exhibited liquid‐like behavior (tan *δ* > 1), similar to that reported by Karasu et al. (2015) and Sereti et al. (2021), and were classified as dilute solutions (Jiang et al. 2021). However, the obtained tan δ values were much lower than those reported for liquid honeys, such as Brazilian honeys (81.5 to 573.0; Silva et al. 2016), owing to the reduced mobility of water molecules in the presence of a high content of α‐d‐glucose monohydrate crystals formed during controlled crystallization in creamed honey processing (Karasu et al. 2015). The formation of this crystalline network results in stronger interactions than those in liquid honeys.

*G*′ and *G*″ were affected (*p* < 0.05) by the interaction between propeller type and agitation speed: at 1 Hz, the *G*′ and *G*″ values of N180 were higher (*p* < 0.05) than those of A180 and N360, whereas the *G*′ and *G*″ values of A360 did not differ (*p* > 0.05) from those of A180 and N360 (Figure 2). This indicated that N180 developed a more strongly interconnected and organized α‐d‐glucose monohydrate crystalline network (Sereti et al. 2021). Kurt et al. (2020) and Ozmen et al. (2025) reported *G*″ values at 100 Hz and 25°C close to 2000 Pa for honeys subjected to controlled crystallization. In the present study, the *G*″ values obtained from the frequency sweep (Figure 2b) were substantially higher, as the test was conducted at 20°C. The results reported by these authors corroborate the influence of the crystallization process on the viscoelastic properties of honey.

**FIGURE 3:**
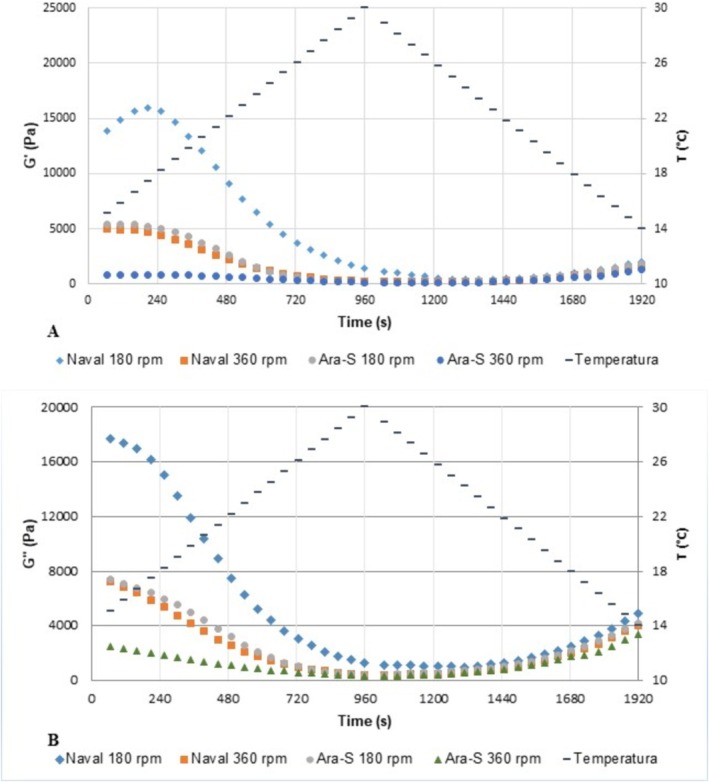
Temperature sweep behavior of the storage modulus (*G*′) (a) and loss modulus (*G*″) (b) of creamed honeys produced using different propeller types and stirring speeds (*n* = 3).

The decline of *G*′ (Figure 3a) and *G*″ (Figure 3b) with increasing temperature indicated the dissolution of glucose crystals, whose solubility temperature is 30°C. Continuous dissolution of the crystalline network releases water molecules, making the system more liquid and homogeneous, and causing a sharp decrease in *G*″ (Figure 3b) (Sereti et al. 2021). Temperature sweep tests demonstrated the low thermal stability of the treatments, as previously reported in other studies (Karasu et al. 2015; Sereti et al. 2021). After the heating–cooling cycle, the final *G*′ values were lower than the initial ones, confirming the structural damage to glucose crystals. Following dissolution, the cooling temperature and time were insufficient to promote recrystallization, as the nucleation and crystal growth stages induced during creamed honey processing did not recur (Karasu et al. 2015). Thus, the product should be stored below 30°C. The heating curve (Figure 3) of the N180 sample exhibited the highest *G*′ and *G*″ values, whereas the A360 sample showed the lowest. A similar trend was observed in the frequency sweep tests, confirming that the propeller type and agitation speed influenced the viscoelastic behavior of the product through variations in the strength of molecular interactions within the α‐D‐glucose monohydrate crystalline network and crystal size (Meixner et al. 2022). After dissolution, the cooling curves (Figure 3) were similar across treatments because the same raw material was used.

### 3.4 Sensory Analysis

The evaluated creamed honey showed good sensory acceptance and purchase intention, differing from those obtained with naturally crystallized honey (CH). The sensory attributes of creamed honeys did not differ significantly among themselves (*p* > 0.05) (Table 4), with mean scores ranging between the hedonic terms “moderately liked” and “liked very much.” Conversely, CH showed a lower acceptance (*p* < 0.05), with mean scores between “slightly disliked” and “moderately liked.” Regarding purchase intention, creamed honey showed higher values (*p* < 0.05) than CH did. For creamed honeys, mean scores ranged between “might buy” and “would certainly buy,” whereas CH obtained mean scores between “would probably not buy” and “might buy.”

**TABLE 4:** Sensory characterization of creamed honeys produced using a mechanical stirrer with different propeller types (Naval and ARA‐S propeller) and stirring speeds (180 and 360 rpm) (*n* = 142).

Treatment^1^, ^2^	Appearance	Flavor	Texture	Overall impression	Purchase intention
N180	7.46 ± 0.13^a^	7.51 ± 0.12^a^	7.46 ± 0.14^a^	7.45 ± 0.11^a^	3.78 ± 0.08^a^
N360	7.23 ± 0.14^a^	7.74 ± 0.11^a^	7.49 ± 0.15^a^	7.64 ± 0.11^a^	3.94 ± 0.09^a^
A180	7.53 ± 0.13^a^	7.72 ± 0.11^a^	7.73 ± 0.12^a^	7.72 ± 0.09^a^	4.08 ± 0.08^a^
A360	7.35 ± 0.15^a^	7.63 ± 0.13^a^	7.63 ± 0.15^a^	7.54 ± 0.13^a^	3.90 ± 0.09^a^
CH	6.67 ± 0.16^b^	6.43 ± 0.16^b^	4.97 ± 0.19^b^	5.94 ± 0.16^b^	2.85 ± 0.10^b^
*p*(*F*)	< 0.001	< 0.001	< 0.001	< 0.001	< 0.001

*Note:* N180: Naval propeller at 180 rpm; N360: Naval propeller at 360 rpm; A180: ARA‐S propeller at 180 rpm; A360: ARA‐S propeller at 360 rpm; CH: crystallized honey; *p*(*F*): probability of the *F*‐test from the Analysis of Variance (ANOVA).

^1^ Means ± Standard Error.

^2^ Means followed by different superscript letters (a,b) differ significantly for the evaluated sensory attribute according to Tukey's test at the 5% significance level.

**FIGURE 4:**
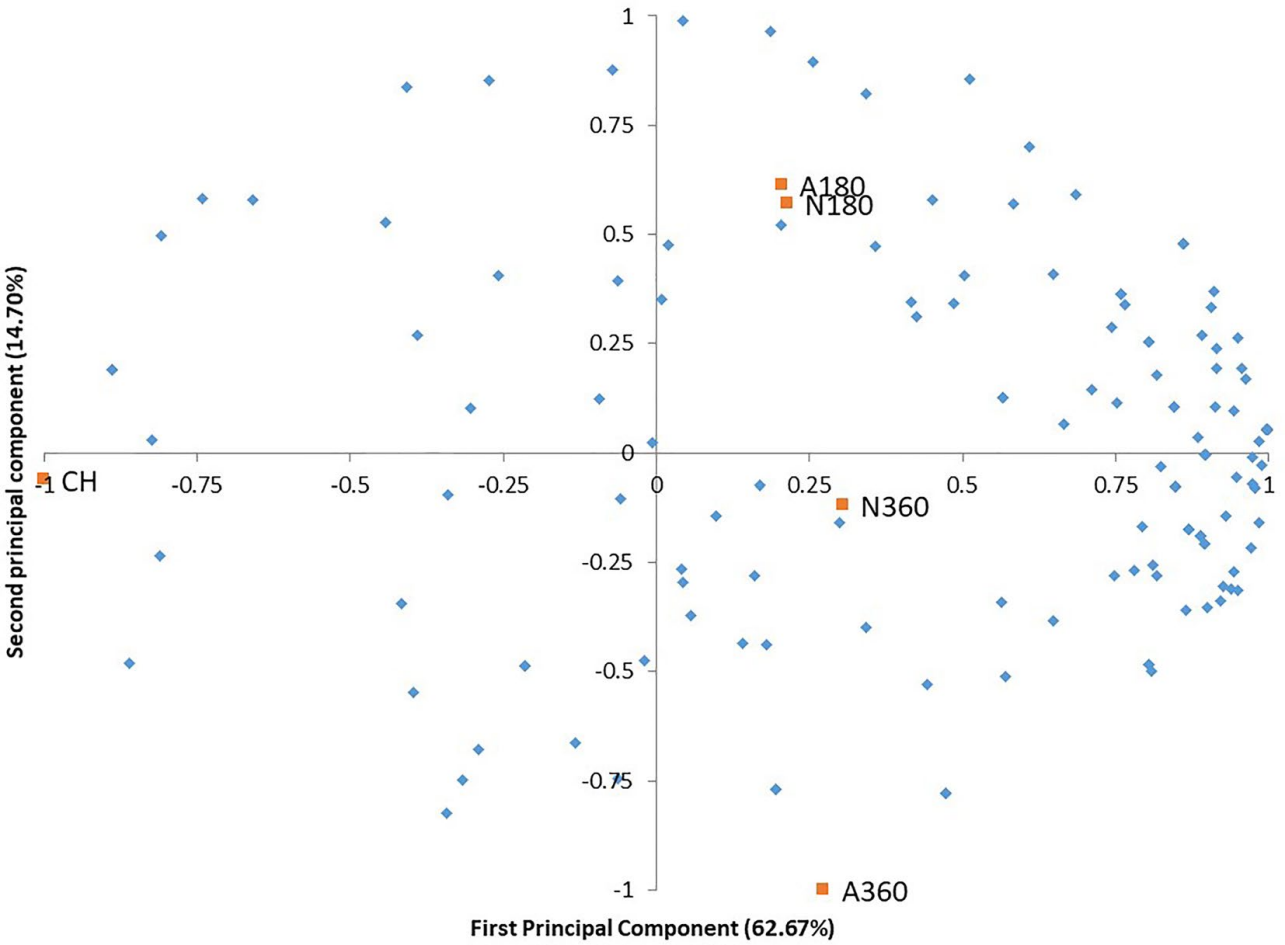
Internal preference map based on overall acceptance scores. N180: Naval propeller at 180 rpm; N360: Naval propeller at 360 rpm; A180: ARA‐S propeller at 180 rpm; A360: ARA‐S propeller at 360 rpm; CH: Crystallized honey.

According to the internal preference map (Figure 4), the first principal component explained 62.67% of the variation in acceptance of the creamed honey samples, whereas the second component explained 14.70%. Together, they accounted for 77.37% of the total variance, which was considered sufficient to discriminate the samples regarding overall acceptance (Figure 4). The internal preference map confirmed the high acceptance of creamed honey samples. A greater concentration was observed in quadrants I and IV, indicating a preference for samples A180, A360, N180, and N360, which were clustered in the first group. These samples did not differ significantly in terms of overall acceptance. The second group only comprised the control sample (CH), which was the least accepted by consumers.

Similar to the findings in the European markets (Piana et al. 2023), Brazilian consumers also preferred creamed honey over naturally crystallized honey. Therefore, the operational method and processing conditions employed resulted in products with high sensory acceptance and purchase intention, outperforming naturally crystallized honey and representing an alternative to increase the added value of honey.

## 4 Conclusion

Overall, this study investigated the effects of propeller type and stirring speed during the mixing step of macerated crystal seeds with liquid honey during product processing. The physicochemical quality of creamed honey was not substantially affected by the studied factors, and all samples complied with the requirements established by the Codex Alimentarius. Some effects of the evaluated factors on the rheological properties were observed. The propeller type influenced *η*_10_, with the Naval propeller yielding higher *η*_10_ values. Both *G*′ and *G*″ were affected by the interaction between propeller type and agitation speed in the frequency and temperature sweeps. The use of the Naval propeller at 180 rpm resulted in higher *G*′ and *G*″ values in both oscillatory tests. All treatments showed pseudoplastic behavior with a good fit to the power law model, thixotropy as described by the Weltman model, temperature dependence of *η*_10_ as explained by the Arrhenius model, liquid‐like behavior, and low thermal stability of the crystalline structure. All creamed honey samples obtained high sensory acceptance and purchase intention, whereas naturally granulated honey presented inferior sensory quality.

These results demonstrate the potential of creamed honey processing and commercialization to increase the added value of raw honey with moderate to high crystallization rates. Although the propeller type and stirring speed influenced certain rheological properties, these variations did not exert a practical impact on consumer acceptance or purchase intent, indicating that the final product presented satisfactory physicochemical and sensory qualities under any of the studied conditions. Therefore, the choice of mixing conditions during processing should be guided by technical and economic criteria. Furthermore, based on rheological characterization data, designing and selecting suitable processing equipment and establishing product quality controls are possible, providing relevant information for both beekeepers and the honey industry.

Despite the results obtained, future studies should focus on optimizing stirring duration, evaluating long‐term shelf stability under different storage conditions, and developing flavored creamed honey variants to expand product diversification.

## Author Contributions

Conceptualization: Vanelle Maria da Silva, Naiara Barbosa Carvalho, and Robledo de Almeida Torres Filho. Methodology: Vanelle Maria da Silva, Naiara Barbosa Carvalho, and Robledo de Almeida Torres Filho. Investigation, analysis: Letícia Oliveira Almeida Silva, Júlia Flávia Souza Andrade, Yasmim Emanuele Oliveira Marques, Janaina Mota Fiuza da Silva, and Vanelle Maria da Silva. Writing – original draft: Letícia Oliveira Almeida Silva, Júlia Flávia Souza Andrade, Yasmim Emanuele Oliveira Marques, and Janaina Mota Fiuza da Silva. Writing – reviewing and editing: Vanelle Maria da Silva, Naiara Barbosa Carvalho, and Robledo de Almeida Torres Filho.

## Funding

The authors gratefully acknowledge Fundação de Amparo à Pesquisa do Estado de Minas Gerais (FAPEMIG) for funding this research (process APQ‐03935‐22) and both Conselho Nacional de desenvolvimento científico e tecnológico (CNPq) and FAPEMIG for supporting undergraduate research scholarships.

## Conflicts of Interest

The authors declare no conflicts of interest.

## Data Availability

The data that support the findings of this study are available on request from the authors.
